# Supplementary material for: Response surface methodology reveals proportionality effects of plant species in conservation plantings on occurrence of generalist predatory arthropods
Source: PLoS One. 2020 Apr 29;15(4):e0231471. doi: 10.1371/journal.pone.0231471 (PMC7190168; doi:10.1371/journal.pone.0231471)
Supplement: S1 Table — (PDF) [file pone.0231471.s001.pdf]

Spring 2017 Experiment

Key to Abbreviations: Em = *Euphorbia milii*, crown of thorns; Pl = *Phaseolus lunatus*, lima beans; Eh = *E. heterophylla*, wild poinsettia; X = damaged sticky card trap

Replicate dates: R1: June 2-7; R2: June 7-10; R3: June 10-13; R4: June 13-16; R5: June 16-20; R6: June 20-23

Coccinellids

Proportion of plant spp in mix			Replicate							
Em	Pl	Eh	R1	R2	R3	R4	R5	R6	TOTAL	MEAN
0.67	0.17	0.17	0	2	6	1	6	8	23	3.8
0	1	0	0	2	23	2	29	7	63	10.5
0	0	1	4	3	1	0	3	2	13	2.2
0	0.5	0.5	2	2	3	5	11	0	23	3.8
1	0	0	1	3	0	3	7	0	14	2.3
0.17	0.67	0.17	6	9	22	17	39	13	106	17.7
0.17	0.17	0.67	0	3	8	1	10	6	28	4.7
0.33	0.33	0.33	0	3	1	0	7	14	25	4.2
0.5	0.5	0	2	3	4	2	30	20	61	10.2
0	1	0	1	8	10	7	13	4	43	7.2
1	0	0	2	1	1	0	3	3	10	1.7
0.5	0.5	0	2	6	2	7	8	5	30	5.0
0	0	1	1	1	2	1	1	1	7	1.2
0.5	0	0.5	1	3	1	4	2	0	11	1.8
0	0	0	1	0	2	2	6	1	12	2.0
0	0	0	0	0	1	2	0	2	5	0.8

Blattella asahinai

Proportion of plant spp in mix			Replicate							
Em	Pl	Eh	R1	R2	R3	R4	R5	R6	TOTAL	MEAN
0.67	0.17	0.17	5	3	5	X	5	1	19	3.8
0	1	0	3	12	19	3	11	6	54	9.0
0	0	1	2	8	8	9	19	9	55	9.2
0	0.5	0.5	5	9	13	14	12	3	56	9.3
1	0	0	3	3	4	X	2	0	12	2.4
0.17	0.67	0.17	30	18	13	11	28	15	115	19.2
0.17	0.17	0.67	5	7	16	14	8	7	57	9.5
0.33	0.33	0.33	7	30	36	X	38	37	148	29.6
0.5	0.5	0	10	20	7	9	11	8	65	10.8
0	1	0	2	19	19	4	7	3	54	9.0
1	0	0	4	4	7	2	8	1	26	4.3
0.5	0.5	0	5	27	12	6	21	9	80	13.3
0	0	1	4		17	1	14	11	47	9.4
0.5	0	0.5	10	11	8	5	12	6	52	8.7
0	0	0	5	1	2	4	4	3	19	3.2
0	0	0	3	2		1	1	0	7	1.4

Predatory Hemipterans

Proportion of plant spp in mix			Replicate							
Em	Pl	Eh	R1	R2	R3	R4	R5	R6	TOTAL	MEAN
0.67	0.17	0.17	0	0	4	1	5	1	11	1.8
0	1	0	1	0	2	1	1	0	5	0.8
0	0	1	3	0	0	0	1	0	4	0.7
0	0.5	0.5	4	0	0	0	2	0	6	1.0
1	0	0	5	0	5	5	12	0	27	4.5
0.17	0.67	0.17	0	2	1	0	3	1	7	1.2
0.17	0.17	0.67	0	0	4	2	0	1	7	1.2
0.33	0.33	0.33	2	1	1	0	4	1	9	1.5
0.5	0.5	0	2	1	1	1	0	1	6	1.0
0	1	0	0	3	0	1	0	1	5	0.8
1	0	0	2	0	1	0	2	2	7	1.2
0.5	0.5	0	3	1	0	0	1	0	5	0.8
0	0	1	0	1	1	0	1	2	5	0.8
0.5	0	0.5	5	0	1	1	4	0	11	1.8
0	0	0	1	0	1	0	0	0	2	0.3
0	0	0	0	0	3	0	2	1	6	1.0
