# Supplementary material for: Response surface methodology reveals proportionality effects of plant species in conservation plantings on occurrence of generalist predatory arthropods
Source: PLoS One. 2020 Apr 29;15(4):e0231471. doi: 10.1371/journal.pone.0231471 (PMC7190168; doi:10.1371/journal.pone.0231471)
Supplement: S2 Table — (PDF) [file pone.0231471.s002.pdf]

Summer 2017 Experiment

Key to Abbreviations: Em = *Euphorbia milii*, crown of thorns; Cf = *Chamaecrista fasciculata*; Fe = *Fagopyrum esculentum*

Replicate dates: R1: Aug. 1-4; R2: Aug. 4-8; R3: Aug. 8-11; R4: Aug.11-15; R5: Aug. 15-18; R6: Aug. 18-22; R7: Aug 22-25

Coccinellids

Proportion of plant spp in mix			Replicate								
Fe	Cf	Em	R1	R2	R3	R4	R5	R6	R7	TOTAL	MEAN
0	0	0	1	0	1	1	0	1	0	4	0.6
0	0	0	0	0	0	1	2	0	2	5	0.7
1	0	0	3	2	3	1	1	0	0	10	1.4
1	0	0	2	3	1	3	0	1	0	10	1.4
0	0	1	2	5	0	0	0	1	0	8	1.1
0	0	1	4	3	2	3	2	1	1	16	2.3
0	1	0	4	0	1	5	3	5	0	18	2.6
0	1	0	2	7	0	1	0	1	1	12	1.7
0.33	0.33	0.33	4	1	0	1	1	2	0	9	1.3
0.5	0	0.5	2	2	1	0	0	2	0	7	1.0
0.5	0.5	0	1	2	1	2	2	2	1	11	1.6
0.5	0.5	0	3	1	2	0	2	1	0	9	1.3
0	0.5	0.5	3	0	1	3	2	0	1	10	1.4
0.67	0.17	0.17	0	4	0	1	1	3	1	10	1.4
0.17	0.17	0.67	2	1	1	1	3	2	2	12	1.7
0.17	0.67	0.17	6	5	1	1	1	0	1	15.0	2.1

Blattella asahinai

Proportion of plant spp in mix			Replicate								
Fe	Cf	Em	R1	R2	R3	R4	R5	R6	R7	TOTAL	MEAN
0	0	0	4	5	3	1	2	0	3	18	2.6
0	0	0	1	0	1	1	0	0	0	3	0.4
1	0	0	3	3	2	0	1	1	0	10	1.4
1	0	0	1	0	1	2	0	0	0	4	0.6
0	0	1	2	2	1	0	2	2	0	9	1.3
0	0	1	3	8	5	10	8	4	1	39	5.6
0	1	0	2	3	0	1	1	1	1	9	1.3
0	1	0	2	4	2	3	1	0	3	15	2.1
0.33	0.33	0.33	5	6	4	3	4	5	3	30	4.3
0.5	0	0.5	3	10	1	2	3	0	2	21	3.0
0.5	0.5	0	3	5	3	0	5	6	1	23	3.3
0.5	0.5	0	1	2	0	0	1	1	0	5	0.7
0	0.5	0.5	5	1	0	2	1	2	4	15	2.1
0.67	0.17	0.17	4	5	4	4	0	2	1	20	2.9
0.17	0.17	0.67	2	1	0	1	1	0	1	6	0.9
0.17	0.67	0.17	3	2	1	0	1	1	1	9	1.3

Predatory Hemipterans

Proportion of plant spp in mix			Replicate								
Fe	Cf	Em	R1	R2	R3	R4	R5	R6	R7	TOTAL	MEAN
0	0	0	0	1	1	2	0	2	0	6	0.9
0	0	0	3	0	1	5	5	0	0	14	2.0
1	0	0	3	5	1	6	3	1	0	19	2.7
1	0	0	7	1	0	5	4	1	0	18	2.6
0	0	1	9	4	0	7	6	0	0	26	3.7
0	0	1	0	2	0	1	0	6	0	9	1.3
0	1	0	0	4	0	2	4	4	1	15	2.1
0	1	0	3	0	1	2	2	2	0	10	1.4
0.33	0.33	0.33	1	1	0	4	1	4	0	11	1.6
0.5	0	0.5	3	3	0	6	0	0	0	12	1.7
0.5	0.5	0	4	1	0	3	1	1	1	11	1.6
0.5	0.5	0	7	0	0	3	3	2	0	15	2.1
0	0.5	0.5	1	2	1	3	0	1	1	9	1.3
0.67	0.17	0.17	1	2	0	1	0	0	0	4	0.6
0.17	0.17	0.67	2	0	0	1	2	5	0	10	1.4
0.17	0.67	0.17	0	0	1	3	0	2	1	7	1.0
