# Supplementary material for: Response surface methodology reveals proportionality effects of plant species in conservation plantings on occurrence of generalist predatory arthropods
Source: PLoS One. 2020 Apr 29;15(4):e0231471. doi: 10.1371/journal.pone.0231471 (PMC7190168; doi:10.1371/journal.pone.0231471)
Supplement: S3 Table — (PDF) [file pone.0231471.s003.pdf]

Summer 2018 Experiment

Key to Abbreviations: Em = *Euphorbia milii*, crown of thorns; Pu = *Portulaca umbraticola*; Fe = *Fagopyrum esculentum*

Replicate dates: R1: July 16-20; R2: July 20-23 R3: July 23-27; R4: July 27-30; R5: July 30-Aug. 3; R6: Aug. 3-6; R7: Aug. 6-10

Coccinellids

Proportion of plant spp in mix			Replicate								
Fe	Pu	Em	R1	R2	R3	R4	R5	R6	R7	TOTAL	MEAN
0	0	0	1	0	0	0	0	1	0	2	0.3
0	0	0	1	0	0	0	0	0	0	1	0.1
1	0	0	1	0	1	0	0	0	1	3	0.4
1	0	0	1	0	1	0	0	0	0	2	0.3
0	0	1	1	1	0	0	0	1	1	4	0.6
0	0	1	1	0	0	0	0	0	2	3	0.4
0	1	0	4	0	0	1	2	1	3	11	1.6
0	1	0	1	0	1	1	3	1	0	7	1.0
0.33	0.33	0.33	1	0	0	0	0	0	0	1	0.1
0.5	0	0.5	1	2	0	1	0	0	0	4	0.6
0.5	0.5	0	2	0	0	1	0	0	1	4	0.6
0.5	0.5	0	2	0	0	1	0	0	0	3	0.4
0	0.5	0.5	1	1	0	1	0	2	0	5	0.7
0.67	0.17	0.17	0	1	0	0	1	2	0	4	0.6
0.17	0.17	0.67	2	1	1	3	0	1	0	8	1.1
0.17	0.67	0.17	0	1	0	3	3	1	0	8	1.1

Blattella asahinai

Proportion of plant spp in mix			Replicate								
Fe	Pu	Em	R1	R2	R3	R4	R5	R6	R7	TOTAL	MEAN
0	0	0	0	0	0	0	0	0	0	0	0.0
0	0	0	0	0	0	2	3	0	5	10	1.4
1	0	0	0	0	0	0	1	0	3	4	0.6
1	0	0	2	1	1	2	2	0	3	11	1.6
0	0	1	0	0	1	3	4	3	0	11	1.6
0	0	1	2	0	1	0	3	2	2	10	1.4
0	1	0	2	0	1	1	1	1	2	8	1.1
0	1	0	2	1	0	1	7	3	1	15	2.1
0.33	0.33	0.33	1	0	1	2	9	2	5	20	2.9
0.5	0	0.5	0	0	2	2	6	3	2	15	2.1
0.5	0.5	0	3	0	0	2	2	0	1	8	1.1
0.5	0.5	0	1	0	0	1	4	0	2	8	1.1
0	0.5	0.5	2	0	2	0	0	2	2	8	1.1
0.67	0.17	0.17	2	1	1	3	5	1	2	15	2.1
0.17	0.17	0.67	2	1	0	1	4	1	2	11	1.6
0.17	0.67	0.17	0	0	2	2	3	0	0	7	1.0

Predatory Hemipterans

Proportion of plant spp in mix			Replicate								
Fe	Pu	Em	R1	R2	R3	R4	R5	R6	R7	TOTAL	MEAN
0	0	0	0	0	0	1	1	1	0	3	0.4
0	0	0	0	0	0	0	0	0	0	0	0.0
1	0	0	0	0	0	0	1	0	0	1	0.1
1	0	0	0	0	0	0	0	0	0	0	0.0
0	0	1	1	0	0	0	0	0	0	1	0.1
0	0	1	0	1	0	2	1	0	0	4	0.6
0	1	0	0	0	0	0	1	0	0	1	0.1
0	1	0	3	0	0	1	0	0	1	5	0.7
0.33	0.33	0.33	0	0	0	1	0	0	0	1	0.1
0.5	0	0.5	1	0	0	0	0	0	1	2	0.3
0.5	0.5	0	1	0	0	0	0	0	0	1	0.1
0.5	0.5	0	0	0	0	0	1	0	0	1	0.1
0	0.5	0.5	0	0	0	0	1	0	0	1	0.1
0.67	0.17	0.17	1	0	0	0	0	0	0	1	0.1
0.17	0.17	0.67	0	0	1	0	1	0	0	2	0.3
0.17	0.67	0.17	0	0	0	0	0	0	0	0	0.0
