# Supplementary material for: Response surface methodology reveals proportionality effects of plant species in conservation plantings on occurrence of generalist predatory arthropods
Source: PLoS One. 2020 Apr 29;15(4):e0231471. doi: 10.1371/journal.pone.0231471 (PMC7190168; doi:10.1371/journal.pone.0231471)
Supplement: Attachment — Submitted filename: Response to Reviewers_031920.docx [file pone.0231471.s005.docx]

Dear Dr. Prager:

I have made the corrections suggested by Reviewer 4, which included the title change. I wish to thank you for your patience and helpfulness in completing the review. I also wish to extend my thanks and gratitude to all of the reviewers; their comments and suggestions have greatly improved the manuscript.

Best Regards—Joe Patt
